# A mixed-type intraductal papillary mucinous neoplasm of the pancreas with a histologic combination of gastric and pancreatobiliary subtypes in a 70-year-old woman: a case report

**DOI:** 10.1186/s13256-020-02464-z

**Published:** 2020-09-09

**Authors:** Sawsan Ismail, Rama Darwisho, Mohammed Ali, Maen Haidar, Mohammad Adib Houreih, Zuheir Alshehabi

**Affiliations:** 1 Department of Pathology, Cancer Research Center, Faculty of Medicine, Tishreen University, Lattakia, Syria; 2 Department of Nephrology, Tishreen University Hospital, Lattakia, Syria; 3 Department of Obstetrics and Gynecology, Tishreen University Hospital, Lattakia, Syria; 4 Department of General Surgery, Tishreen University Hospital, Lattakia, Syria; 5 Faculty of Pharmacy, Al-Sham Private University, Lattakia, Syria

**Keywords:** Intraductal papillary mucinous neoplasm, Mixed-type IPMN, Endoscopic retrograde cholangiopancreatography, Gastric and pancreatobiliary subtypes, Distal pancreatectomy

## Abstract

**Background:**

Intraductal papillary mucinous neoplasms are rare papillary pancreatic neoplasms arising from major pancreatic ducts, characterized by duct dilation and mucin secretion. They comprise approximately 1% of all exocrine neoplasms and are classified according to their anatomical sites into main duct-type, branch duct-type, and mixed-type intraductal papillary mucinous neoplasms. Histological examination plays a crucial role in distinguishing and classifying intraductal papillary mucinous neoplasms into gastric, intestinal, pancreatobiliary, and oncocytic subtypes.

**Case presentation:**

We present the case of a 70-year-old Syrian woman who was admitted to our hospital due to an intermittent epigastric pain accompanied by diarrhea and weight loss with a recent diagnosis of diabetes mellitus. Following clinical, laboratory, and radiological examination, distal pancreatectomy involving the body and the tail of the pancreas was performed. Interestingly, histological examination of the resected specimens revealed the diagnosis of a mixed-type intraductal papillary mucinous neoplasm with a unique combination of gastric and pancreatobiliary subtypes.

**Conclusion:**

To the best of our knowledge, the combination of multiple histological subtypes of intraductal papillary mucinous neoplasms has been recorded in a few studies with reference to the challenging histological detection. Herein, we report a rare case with a significant histological combination, highlighting the difficulties in differential diagnosis due to the absence of ancillary techniques, with a brief review on diagnostic methods, histological characteristics and surgical recommendations.

## Background

Intraductal papillary mucinous neoplasms (IPMNs) were first defined by Ohashi *et al.* in 1982 as rare papillary pancreatic neoplasms arising from major pancreatic ducts, characterized by duct dilation and mucin secretion [1]. They comprise approximately 1% of all exocrine neoplasms, and they are more common in older men. These neoplasms are usually asymptomatic in early stages, but multiple cases present with recurrent episodes of pancreatitis, in addition to diabetes and steatorrhea due to pancreatic insufficiency [2].

They are primarily classified as main duct-type (MD-IPMN), branch duct-type (BD-IPMN), and mixed-type IPMN, which is defined as the anatomic combination of both types [3–5]. Also, histological classification depending on the epithelial cells includes gastric, intestinal, pancreatobiliary, and oncocytic subtypes [2, 3, 6]. Herein, we report the case of a 70-year-old woman who was diagnosed with a mixed-type IPMN with a rare combination of gastric and pancreatobiliary subtypes.

## Case presentation

A 70-year-old Syrian woman presented to the gastroenterology department of our hospital in March 2019 due to an intermittent localized epigastric pain for 10 days accompanied by diarrhea, nausea, and weight loss with no fever. Her blood pressure and electrocardiogram results were normal during physical examination., Six days previously, our patient had noticed that her urine was a dark color.

Her medical and surgical history mentioned a recent diagnosis of type 2 diabetes mellitus during a routine check-up examination only 25 days prior to the initial presentation, in addition to a history of a partial thyroidectomy 20 years ago and hip replacement surgery 7 years ago. Her family history included a diagnosis of type 2 diabetes mellitus in her father and brother, while her psychosocial history was unremarkable. Additional files and details of our patient’s surgical history and previous medical investigations before presentation to our institution were not available.

On initial presentation to our hospital, a blood test revealed a white blood cell (WBC) count of 5 × 10^3^/UL, a lymphocyte count of 47%, a neutrophil count of 50%, hemoglobin value of 13.2 g/dl, total bilirubin of 0.8 mg/dl, direct bilirubin of 0.3 mg/dl, amylase of 42 U/L, glucose value of 237 mg/dl, and C-reactive protein (CRP) value of 120. In addition, her thyroid-stimulating hormone (TSH) value was determined to be 8.22 u IU/m (reference range 0.38–4.31 u IU/ml), with normal values of free thyroxine (FT4) and free triiodothyronine (FT3).

**Fig. 1:**
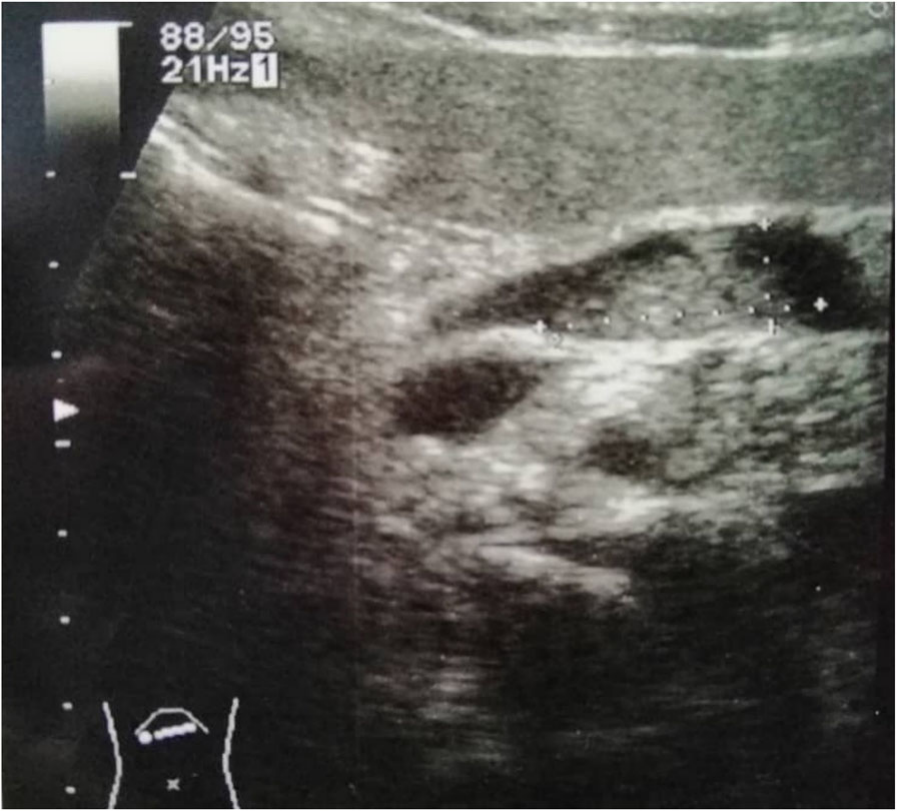
Abdominal ultrasound revealing a cystic dilation in the main pancreatic duct

**Fig. 2:**
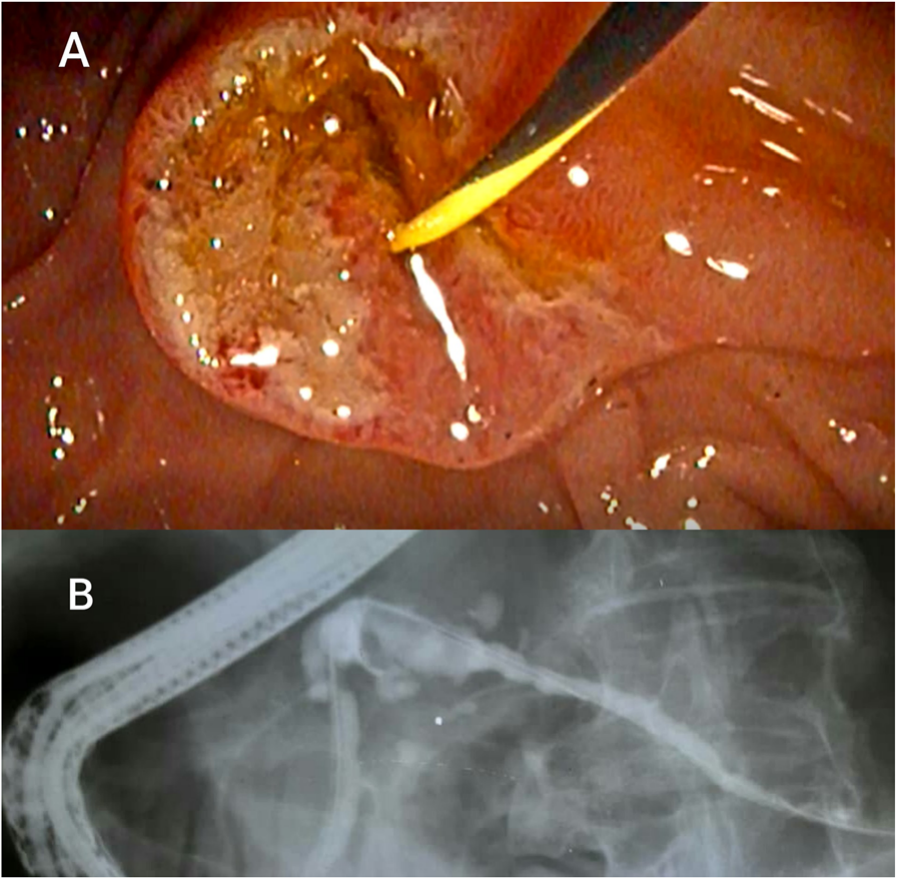
**a** Endoscopic retrograde cholangiopancreatography (ERCP) demonstrating a cystic dilation in the main duct surrounding an irregular-shaped mucinous lesion that was catheterized by bioptome. **b** X-ray image during ERCP. *ERCP* endoscopic retrograde cholangiopancreatography

**Fig. 3:**
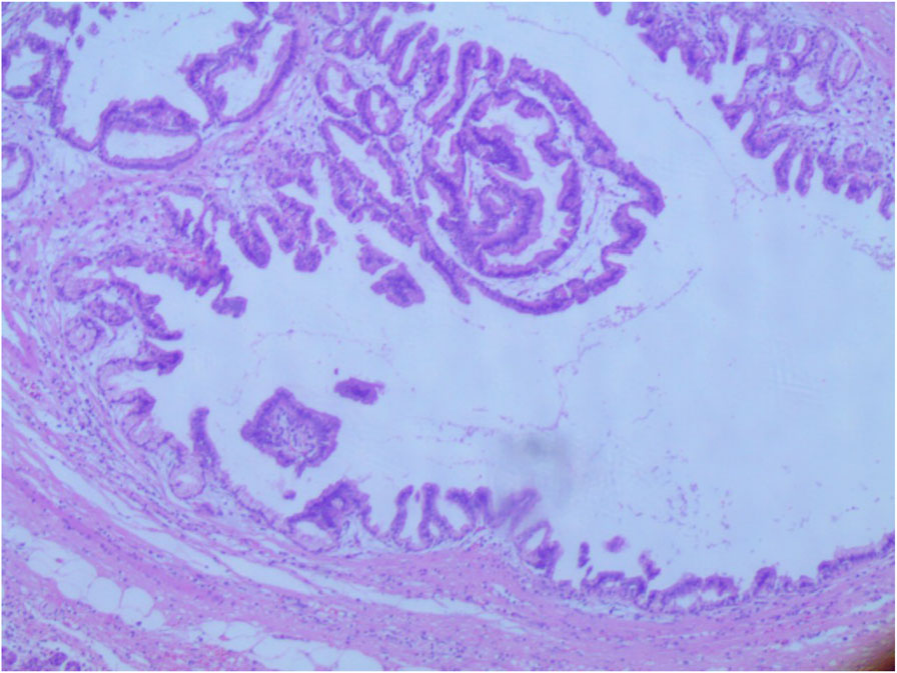
Morphologic features of both pancreatobiliary epithelium (*upper left*) and gastric epithelium (*lower right*) (hematoxylin and eosin (H&E) stain, original magnification ×200). *H&E* hematoxylin and eosin

**Fig. 4:**
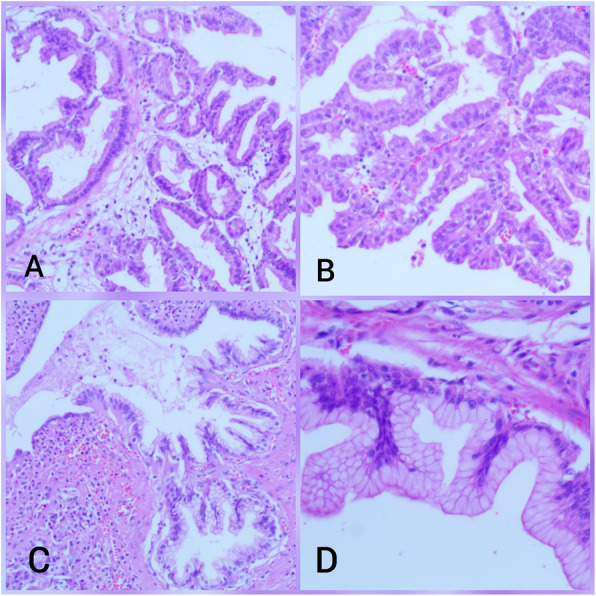
**a** and **b** Complex branching papillae lined by cuboidal cells with moderate amphophilic cytoplasm and hyperchromatic nuclei, demonstrating morphological features of pancreatobiliary epithelium. **c** and **d** Short papillae lined by columnar cells with eosinophilic cytoplasm and basally located nuclei, demonstrating morphological features of gastric epithelium. (hematoxylin and eosin (H&E) stain, **a** original magnification ×200, **b** original magnification ×400, **c** original magnification ×200, **d** original magnification ×600). *H&E* hematoxylin and eosin

**Fig. 5:**
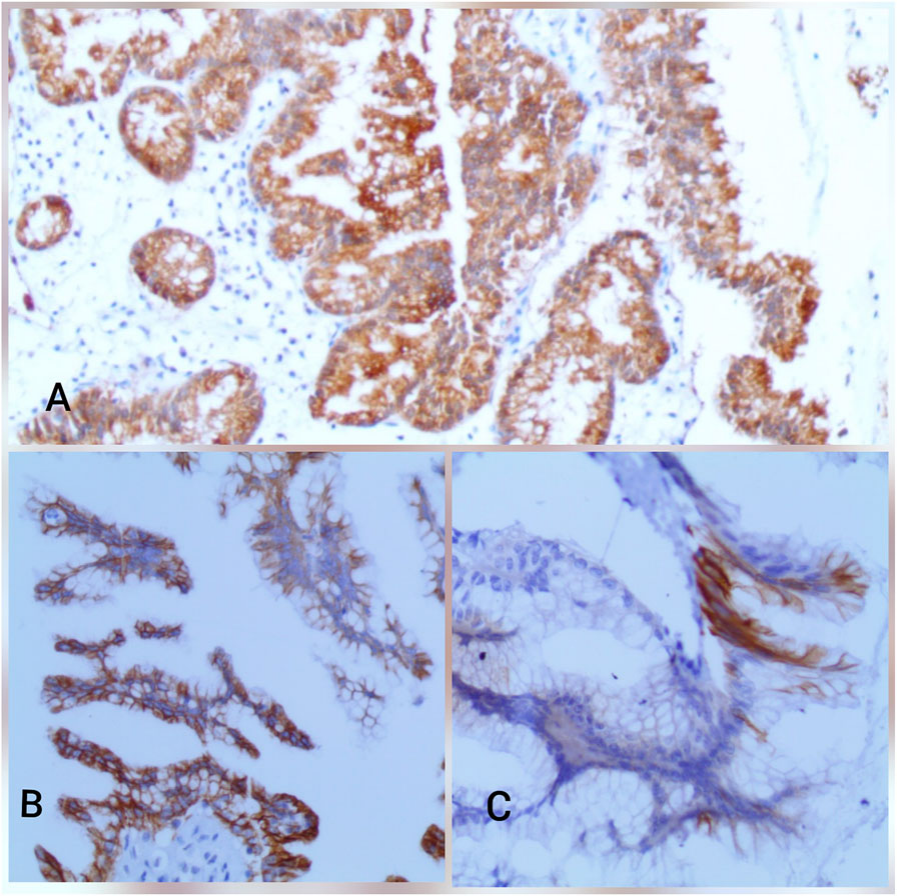
Immunohistochemistry (IHC) of the neoplasm. **a** High positivity for EMA. **b** High positivity for CK7. **c** Low positivity for CK20. *CK* cytokeratin, *EMA* epithelial membrane antigen, *IHC* immunohistochemistry

An abdominal ultrasound revealed a cystic dilation in the main pancreatic duct across the body of the pancreas, and involved an irregular-shaped mass (Fig. 1). Endoscopic retrograde cholangiopancreatography (ERCP) demonstrated a fish-mouth appearance of the ampulla of Vater, which was catheterized by bioptome, and revealed a 15 mm cystic dilation in the main duct surrounding an irregular-shaped mucinous lesion in the connection point between the head and the body of the pancreas, and connected laterally to multiple small cleft-like cysts in the smaller branch ducts (Fig. 2). The primary differential diagnosis according to the radiological findings included intraductal papillary mucinous neoplasm (IPMN) and mucinous cystic neoplasm (MCN), and our patient was then referred to the surgical department and scheduled for a surgical operation. Two days later, our patient underwent distal pancreatectomy involving the body and the tail of the pancreas in addition to a cholecystectomy. The resected specimens consisted of multiple fragmented pieces of tissue from the intrapancreatic duct tumor, in addition to multiple pieces labeled as proximal margins, the gallbladder, and a piece of pancreatic tissue. The histological examination of the tumor revealed complex branching papillae lined by cuboidal cells with moderate amphophilic cytoplasm and enlarged hyperchromatic nuclei resembling cholangiopapillary epithelium with intermediate-grade dysplasia, in addition to short thick papillae lined by mucin-producing columnar cells with eosinophilic cytoplasm and basally located nuclei with low-grade dysplasia resembling gastric foveolae (Figs. 3 and 4). Immunohistochemical (IHC) stainings revealed high positivity for epithelial membrane antigen (EMA) and cytokeratin 7 (CK7), with weak positivity for cytokeratin 20 (CK20) (Fig. 5). However, the aforementioned IHC markers are considered general markers of ductal epithelial cells and unfortunately, additional ancillary techniques including molecular and additional IHC markers were not available in our pathology lab.

Nevertheless, based on detailed examination of the radiological and morphological features with multiple pathological and radiological consultations, the diagnosis was confirmed as a low-grade mixed-type IPMN with a mixed histological combination of gastric and pancreatobiliary subtypes. The rest of the pancreatic tissue was diagnosed as chronic pancreatitis. Although the admitted specimens included pieces of tissue labeled as proximal margins according to the surgical report, margins status could not be assessed by the pathologist due to the fragmentation of the specimens. Nevertheless, there was no evidence of high-grade dysplasia or malignancy. A whole-body computed tomography (CT) scan revealed no suspected lesions in her other organs. After the surgery, blood tests revealed lymphopenia, anisocytosis, high levels of CRP and serum glucose. However, our patient’s test results, except for glucose and TSH, went back to normal 5 days later. No adjuvant therapy was recommended, and our patient was put on levothyroxine (Eltroxin) (due to the high level of TSH), and insulin (Mixtard). According to the monthly blood test results, her TSH levels went back to normal 5 months later, while a moderate elevation in glucose value is still being reported (ranging between 203 and 245 mg/dl up to the time of reporting this case).

**Fig. 6:**
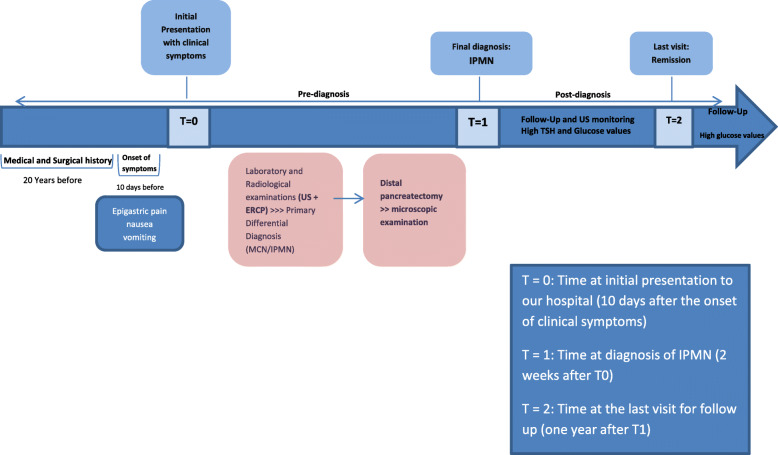
A timeline of our patient’s case.

Furthermore, ultrasonographic monitoring every 6 months was recommended. From the surgical procedure until her last visit in March 2020, our patient has been in a stable condition. A timeline of our patient’s case can be seen in Fig. 6.

## Discussion

IPMNs comprise approximately 21–41% of pancreatic cystic neoplasms and 1–3% of all pancreatic exocrine neoplasms. Although the exact incidence is still unknown due to the absence of symptoms in most cases [2, 3], some patients report atypical symptoms including epigastric pain, weight loss, nausea, vomiting, and jaundice. The majority of cases have been reported as benign with a predominance in 60–70-year-old men [3, 4]. However, our case was symptomatic and reported in a 70-year-old woman.

Many risk factors have been shown to increase the incidence of these neoplasms including diabetes mellitus, as in our case, chronic pancreatitis, family history of pancreatic ductal adenocarcinoma, and mutations in GNAS and KRAS genes. Interestingly, smoking is not reported as a risk factor although it can induce malignancy in IPMNs [5].

According to their anatomical sites, IPMNs are classified into main duct-type (MD-IPMN), which has a higher risk of malignancy, branch duct-type (BD-IPMN), which is associated with multifocal cystic lesions, and mixed-type IPMN, which is a combination of both types [3]. As our case demonstrated a cystic dilation involving the main duct and connected to multiple small cleft-like cysts, the diagnosis was a mixed-type IPMN.

While IPMNs might be accompanied by extra-pancreatic malignant tumors involving the breast, lungs, prostate, colon, and rectum [3–5], CT scan and ultrasound scanning were free of any additional neoplasms in our patient.

Abdominal CT scan has been historically considered the most common radiological method used in diagnosing IPMN. However, ERCP is considered more reliable for early diagnosis and demonstrating detailed features of the ductal system [6, 7]. In our patient, ERCP enabled us to determine the anatomical type of the neoplasm and the size of the cystic dilation, which referred to a higher risk of malignancy.

Studies reveal that both CT and magnetic resonance imaging have equal accuracy in staging malignancy of pancreatic ductal neoplasms [6, 8], Magnetic resonance cholangiopancreatography (MRCP) appears to have more sensitivity than ERCP to demonstrate internal septations and multiple focal cysts according to International Consensus Guidelines 2012 [8], it is also recommended to be used in the follow-up. Also, endoscopic ultrasound (EUS) elastography and confocal laser endomicroscopy are new methods to diagnose IPMNs but need further evaluation before putting in use [6, 7, 9].

Furthermore, EUS is highly preferable as an imaging method for pancreatic cystic neoplasms as it can distinguish IPMNs anatomical types, and demonstrate features of suspected malignancy, in addition to the ability to perform a guided fine-needle aspiration for further evaluation [9].

Histologically, a considerable heterogeneity is noticed in the epithelium by identifying four histological subtypes of the precursor component: Gastric subtype, which is characterized by thick short papillae lined by columnar cells with eosinophilic cytoplasm, basally located nuclei, and strong positivity for MUC5AC. This subtype represents the most common subtype and is usually associated with the BD-type in contrast to our case. Other histological subtypes include the intestinal subtype, which is usually associated with the MD-type and resembles intestinal villous neoplasms with columnar cells, pseudostratified hyperchromatic nuclei and strong positivity for MUC2; pancreatobiliary subtype, which is a rare subtype characterized by complex papillae lined by cuboidal cells with amphophilic cytoplasm, hyperchromatic nuclei, marked atypia, and strong positivity for MUC1; and oncocytic subtype, which presents with branching papillae lined by oncocytic cells with intracellular lumina and focal positivity for MUC1 [10–12]. Interestingly, although the oncocytic subtype is considered a histological subtype of IPMNs according to the World Health Organization classification of pancreatic tumors in 2010 [13], recent studies suggest defining it as a separate type due to distinct morphological and molecular features [14].

Nevertheless, our case showed a rare combination of gastric and pancreatobiliary subtypes in a mixed-type IPMN that was diagnosed based on assessing morphological features with hematoxylin and eosin (H&E) stainings only due to economic restrictions, as the aforementioned IHC stainings were not available.

Despite the above, at the current time, it is not likely that histological subtypes have an influence on decided clinical management in non-invasive neoplasms like in our case, while they have a significant prognostic implication in invasive carcinoma [10, 11].

Differential diagnosis includes many lesions, most commonly are MCNs, serous cystadenoma, pseudocysts, and chronic pancreatitis. Many factors play a crucial role in establishing the diagnosis including the age of the patient, radiological findings, clinical presentation, anatomical location, and morphological features [15].

MCNs are spherical cysts with peripheral calcifications, an ovarian-type stroma, and variable-thickened septations. They are more common in young women, and they usually arise in the body and the tail of the pancreas without connecting to the ductal system.

Serous cystadenomas tend to affect older women and they are usually located in the head of the pancreas accompanied by multiple microcysts with a honeycomb-like pattern, central calcification, a thin peripheral wall, and a non-mucinous clear fluid. Also, the absence of septations, mural nodules and the lack of an epithelial lining, as well as the presence of necrotic hemorrhagic debris increases the possibility of pseudocysts [15, 16]. Regarding our case, the absence of ovarian-type stroma, and the connection to the ductal system of a mucinous neoplasm in an older patient in addition to other radiological and morphological features were all crucial clues to support the diagnosis.

Genetically, next-generation sequencing revealed that the presence of IPMNs correlates with multiple genetic mutations including GNAS mutations, which are considered the most common and specific alteration in this neoplasm. Also, an activation in G-protein signaling seems to be associated with the progression of IPMNs, while the expression of phosphorylated substrates of protein kinase A contributes to the pathological grade of this neoplasm [17].

Regarding surgical recommendations, researchers have different points of view toward IPMN enucleation because, on one hand, it can preserve the pancreatic function for a long time as most of the parenchyma is preserved. On the other side, cysts may be ruptured during the procedure, which leads to peritoneal seeding. Furthermore, postoperative complications like pancreatic fistula are commonly noticed. Thus, regional pancreatectomy as a curative procedure is the main challenge, and researchers have discussed frozen section analysis as an important component of the surgery [18, 19]. Thus, it is crucial to select the ideal procedure for non-invasive IPMNs considering short- and long-term results, in addition to postoperative complications.

International guidelines for IPMNs treatment include pancreaticoduodenectomy, distal pancreatectomy, or total pancreatectomy with lymph node segmentation and malignancy-free margins [19, 20].

Overall, invasive IPMNs were found to have the best response to pancreaticoduodenectomy, while non-invasive IPMNs of the body and the tail respond to distal pancreatectomy, and finally, patients with diffused IPMNs or IPMNs that extend through the body and tail undergo total pancreatectomy [19, 20]. As our patient was diagnosed with a non-invasive low-grade IPMN, distal pancreatectomy involving the body and the tail of the pancreas was reliable. Unfortunately, fragmentation of the resected specimen prevented accurate assessing of margins status.

One study revealed that invasive IPMNs have recurrence rates of almost 67–91% with a 5-year survival rate of 40–60%, while non-invasive IPMNs are found to have almost no recurrence with a survival rate up to approximately 100% [11, 19, 20]. Our patient has been in a stable condition with no detected recurrence so far. However, as she has been diagnosed with the mixed-type IPMN, which has a higher risk than BD-IPMN of transforming into malignancy, in addition to the presence of multiple risk factors including the large dilation of the main duct (> 1 cm) and the symptoms of jaundice and diabetes; surgical resection followed by regular monitoring is essential [6, 20].

## Conclusions

In conclusion, our case demonstrated a rare histological combination that represented a hard diagnostic challenge due to the difficulties in differential diagnosis and the lack of proper immunohistochemical and molecular techniques. However, with clinical correlation, accurate observation of radiological and morphological findings, as well as reliable surgical management, we were able to confirm the diagnosis.

## Data Availability

Not applicable.

## Abbreviations

BD-type: Branch duct type
CK: Cytokeratin
CRP: C-reactive protein
CT: Computed tomography
EMA: Epithelial membrane antigen
ERCP: Endoscopic retrograde cholangiopancreatography
EUS: Endoscopic ultrasound
FT3: Free triiodothyronine
FT4: Free thyroxine
H&E: Hematoxylin and eosin
IHC: Immunohistochemistry
IPMN: Intraductal papillary mucinous neoplasm
MCN: Mucinous cystic neoplasm
MD-type: Main duct type
MRCP: Magnetic resonance cholangiopancreatography
TSH: Thyroid-stimulating hormone
WBC: White blood cell
